# Energy Costs of Catfish Space Use as Determined by Biotelemetry

**DOI:** 10.1371/journal.pone.0098997

**Published:** 2014-06-04

**Authors:** Ondřej Slavík, Pavel Horký, Libor Závorka

**Affiliations:** 1 Department of Zoology and Fisheries, Faculty of Agrobiology, Food and Natural Resources, Czech University of Life Sciences Prague, Prague, Czech Republic; 2 Department of Zoology, University of Gothenburg, Göteborg, Sweden; The University of Wollongong, Australia

## Abstract

Animals use dispersed resources within their home range (HR) during regular day-to-day activities. The high-quality area intensively used by an individual, where critical resources are concentrated, has been designated as the core area (CA). This study aimed to describe how animals utilize energy in the HR and CA assuming that changes would occur according to the size of the used areas. We observed energetic costs of space use in the largest European freshwater predator catfish, *Silurus glanis*, using physiological sensors. Catfish consumed significantly more energy within the CA compared to the rest of the HR area. In addition, energetic costs of space use within a large area were lower. These results generally indicate that utilization of larger areas is related to less demanding activities, such as patrolling and searching for new resources and mates. In contrast, fish occurrence in small areas appears to be related to energetically demanding use of spatially limited resources.

## Introduction

The size of the home range (HR) used by animals during regular day-to-day activities [1] to satisfy their energy needs depends on the quality and availability of resources. Animals search for dispersed resources throughout the HR [2], the size of which can be larger than predicted by energy needs [3], [4] reflecting, for example, low habitat productivity, in which the energy needs of the resident animals are poorly satisfied [5], [6], and/or competition with neighbors for resources within the HR [7]–[9]. An unbalanced distribution of resources within the HR results in a disproportional use of the HR [10]. The preferred area of intensive use [11] or the most concentrated [12], [13] within the HR is referred to as the “core area (CA)” [14] and is where individuals spend most of their time[15]. The CA contains critical resources for species [16], representing the highest quality environment [17] and ensuring the best fitness for residents [18]. The size of the CA and HR are mutually correlated, and both areas display an inverse relationship with available food resources [19]. The CA is frequently reported as the most important part of the HR [17], [15]; thus, we assumed that an important amount of energy spent by an individual would occur within the CA and that the changes in energetics would occur according to the size of the used areas. To test our assumptions, we used the radio biotelemetry method. Specimens of the European catfish *Silurus glanis* (L. 1758), a large freshwater predator, were equipped with physiological sensors measuring energetics of an individual [20]. The correlation between electromyogram (hereafter EMG) records and animal behavior including fish is well documented [20]. EMG records were used to describe the movement activity of fish [21], reproductive behavior [22], a relationship between density and energetic expenditures of individuals in aquaculture [23], parental care [24], stress during transport [25], territorial defense [26] or contact between familiar and unfamiliar individuals [27]. The catfish can achieve a body weight of more than 100 kg [28], hunt aquatic and terrestrial vertebrates [29] and display individually characteristic energy costs of movement activity [30]. The catfish was chosen for the study because they show high site fidelity [31] enabling to determine the HR and CA of specimens [26].

## Materials and Methods

### a) Study area

The study was conducted on the Berounka and Elbe Rivers in the Czech Republic. The study sites were located in stretches of river characterized as lowland rivers with a gradual slope. These particular sites were chosen because of native catfish occurrence. A detailed description of the study sites can be found in [32] and [26] for the Berounka River (the river stretch studied was 3.5 km long; 49°55′N; 14°14′E; 49°56′N; 14°17′E) and the Elbe River (the river stretch studied was 20 km long; 50°09′N; 15°48′E; 50°02′N; 15°46′E), respectively. No special permit is required for the field work; the species is not protected by legislation in force. The observation was announced to competent authorities that were: Elbe River Authority and Vltava River Authority, River Management Authorities, state enterprises, and to Czech Anglers Club, civic association, Fish Management Authority.

### b) Fish capture and tagging

A total of 20 individuals (16 from the Berounka River and four from the Elbe River) were captured through electrofishing (650 V, 4 A, pulsed DC). The surgery was performed under 2-phenoxy-ethanol (0.2 ml l^−l^; Merck KGaA; www.merck.com) and all efforts were made to minimize suffering. The fish were weighed and measured (Table 1). The mean weight and length of the captured fish were 20.5 kg (range: 5.5–64.5 kg) and 1.34 m (range: 0.83–2.16 m) for the Berounka River and 17.5 kg (range: 4.4–28.5 kg) and 1.29 m (range: 0.85–1.62 m) for the Elbe River. Electromyogram (EMG) transmitters (CEMG2–R11–25, mass 12 g in air, 11.49 mm, with an operational life of c. 71 days; Lotek; www.lotek.com) were implanted in the body cavity of each fish through a mid-ventral incision that was closed by three separate stitches using a sterile braided absorbable suture (Ethicon coated VICRYL). The two electrodes of the surgically implanted EMG transmitters were positioned in the red aerobic musculature below the lateral line on the left side of the fish. The distance between the electrodes ranged from 10 to 12 mm [33]. The individuals equipped with the EMG transmitters were kept in cages immersed in the river stretch for c. 60 min after implantation of the transmitters. They were then released at or near the point of capture.

**Table 1:** The average values of the core area (CA), EMG within CA (EMG_CA_), Home Range periphery area (HRp) and EMG within HRp (EMG_HRp_) for particular observed catfish individuals.

Fish ID	Weight (kg)	Length (m)	River	CA (m^2^)	EMG_CA_	HRp (m^2^)	EMG_HRp_
25	15.5	1.32	Berounka	755	8.29	2 511	8.19
27	8.5	0.98	Berounka	906	15.99	3 009	15.84
28	9.8	1.05	Berounka	2 433	6.03	8 082	6.24
29	14.6	1.31	Berounka	415	11.98	1 377	11.85
30	7.5	0.87	Berounka	273	7.88	907	7.68
31	9.5	1.07	Berounka	2 054	13.57	6 823	13.17
32	16.5	1.36	Berounka	2 423	12.14	8 048	12.17
33	26.7	1.64	Berounka	302	4.59	1 005	4.92
34	18.5	1.42	Berounka	102	15.68	340	15.63
35	56.5	1.98	Berounka	6 733	11.43	22 365	12.21
36	64.5	2.16	Berounka	4 790	20.95	15 914	20.63
37	11.2	1.12	Berounka	759	7.13	2 523	7.07
38	13.9	1.25	Berounka	287	16.22	954	15.52
39	19.5	1.44	Berounka	6 210	4.55	20 631	4.56
40	29.8	1.71	Berounka	1 409	26.91	4 681	26.41
41	5.5	0.83	Berounka	932	12.21	3 097	12.28
100	4.4	0.85	Elbe	4 014	6.17	13 337	5.27
101	14.8	1.28	Elbe	2 402	6.11	7 982	5.94
102	28.5	1.62	Elbe	46 511	7.27	154 510	6.44
103	22.3	1.39	Elbe	1 487	14.67	4 941	14.93

### c) Sampling procedures

The fish were monitored from 1 March to 31 May 2006 (Elbe River) and from 3 August to 13 October 2009 (Berounka River). All individuals were tracked weekly to check their positions in the river. In addition, a group of randomly selected individuals (four to eight depending on the tracking conditions) was subsequently tracked during a 48 h cycle with two radio receivers (Lotek SRX_600 W31) and a three-element Yagi antenna equipped with a compass. The positions of the fish were determined during 16 subsequent 3 h intervals using a GPS. The data on individual fish movements were transferred from the GPS to a PC and were then analyzed with the aid of Map Source v5.3 (Garmin Ltd; www.garmin.com). A computer program was developed to obtain the position coordinates of the fish and to subsequently plot the coordinates on a map with the biangulation method proposed by [34]. The EMG-coded transmitters allowed multiple transmitters to be monitored simultaneously at the same frequency. Each transmitter had a unique code to identify each fish. The voltage corresponding to muscle activity was rectified and sampled during a 5 s period. The average value over this period was then determined, and an activity level was assigned that ranged from 0 to 50 [33]; these values are henceforth termed EMG values. A set of ten EMG values (5 s burst rate) was obtained three times for all tagged individuals during each 3 h interval. To obtain information on the resting activity levels for standardizing data, the EMG transmitters were individually calibrated for each fish. The resting values were measured twice: first from the anaesthetized fish and prior to release and second from when the fish were held in cages. The average of all recorded EMG resting signals was subsequently considered the baseline value for the particular fish (the ‘baseline EMG value’; [33]).On both rivers, the accuracy of the fish position determination was estimated to be ±3 m according to a calibration procedure performed with a tag located on the riverbed. The calibration was repeated ten times, and the observer did not know the position of the tag.

### d) Data analysis

Schoener index [35] and Swihart & Slade index [36] were computed to test for autocorrelation of successive catfish locations prior to the HR calculations. Locations were suggested to be autocorrelated in several cases. Such data were treated according to Swihart and Slade [37] and the inappropriate locations were removed. The size of the catfish HR was determined using the fixed kernels with least squares cross validation to estimate the smoothing bandwidth [38]. The 50% contour was used to delineate the CA area [12] and the 95% contour to estimate the entire HR area. The difference between the entire HR and CA areas was termed ‘HR periphery’. Bivariate variable describing whether fish was present in the CA or HR periphery was termed ‘fish position’. Each final EMG value included in the subsequent analysis was equal to the recorded EMG value minus the baseline EMG value. The final EMG values were used as general descriptors of energy costs of space use. To ensure that the individual EMG analyses were independent of weight, relative EMG/weight ratios were calculated by dividing each final EMG value by fish weight [30], [39]. ‘Energy costs of space use’ was separately determined in the CA and in the HR periphery as the mean of all recorded EMG/weight ratio values within the catfish ‘used area’.

### e) Statistical analyses

The statistical analyses were performed using the SAS software package (SAS Institute Inc., version 9.2, www.sas.com). The energetic data were analyzed using a linear mixed model (LMM) with random factors (PROC MIXED). The data were transformed for normality prior to the LMM analyses if necessary. The random factors were used to account for the repeated measures collected for the same experimental units (individual fish) across the duration of the experiment in particular rivers. The significance of each exploratory variable was assessed using an F-test. The differences between the classes were tested with a t-test, and a Tukey–Kramer adjustment was used for multiple comparisons. The degrees of freedom were calculated using the Kenward–Roger method [40].

### Ethics statement

The work was prepared and conducted according to valid legislative regulations (Law no. 246/1992, § 19, art. 1, letter c); the permit was subjected to O. Slavík, qualified according to Law no. 246/1992, § 17, art. 1; permit no. CZ00167. All field sampling including EMG transmitter implantation was carried out with the relevant permissions from the Departmental Expert Committee for authorization experimental project of the Ministry of Environment of the Czech Republic (permit no. 26758/ENV/10-1092/620/10-PP6, registered by the Ministry of Environment of the Czech Republic). The study did not involve endangered or protected species.

## Results

For the analysis in this study, were used 2 284 records of fish positions and 22 840 EMG values. The average size of the CA was 6784 m^2^ (range 25–392 622 m^2^) and the average size of the HR periphery was 22 538 m^2^ (range 84–1 304 269 m^2^). More details according to the individual HR size variation can be found in Table 1.

**Figure 1:**
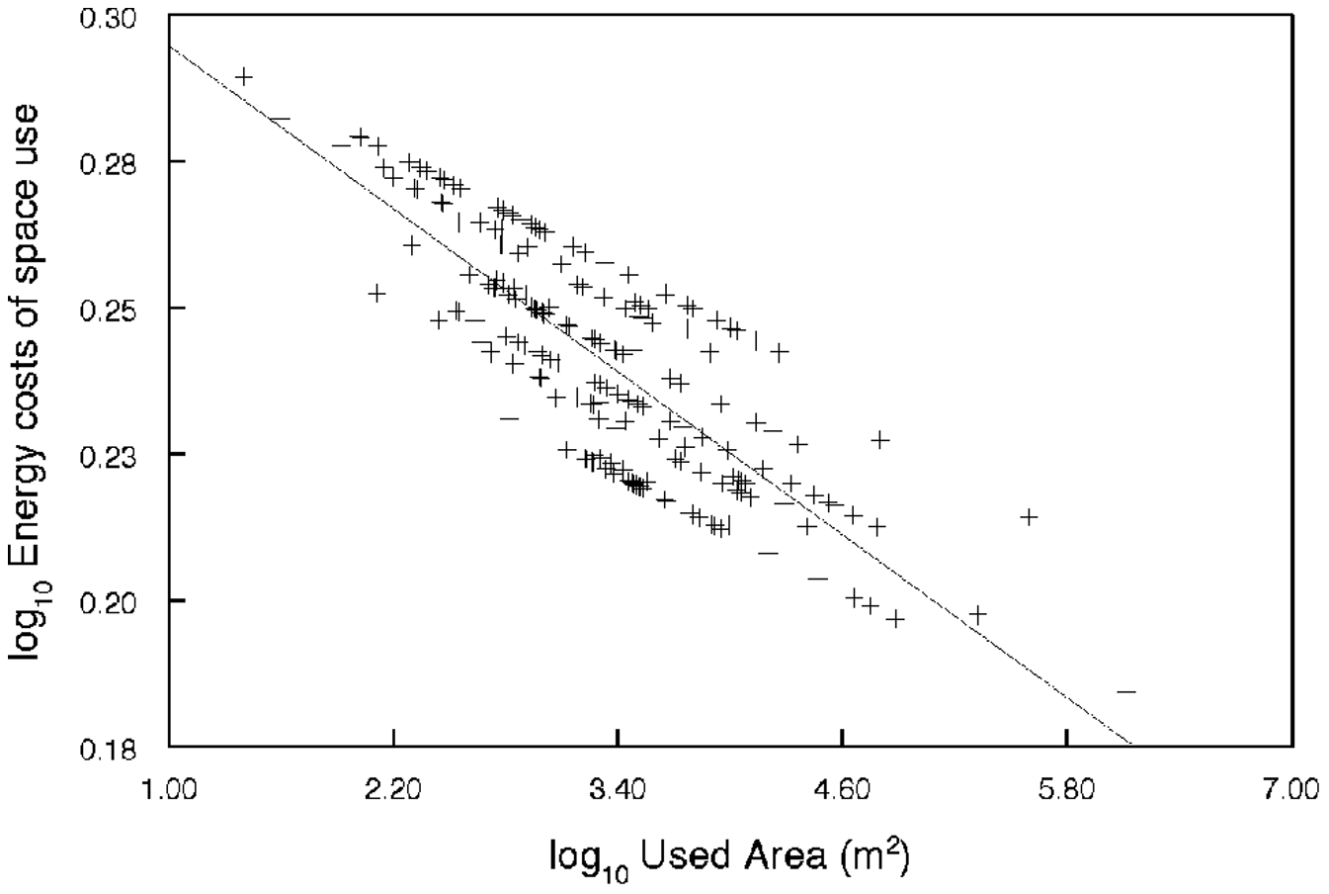
The relationship between energy costs of space use and the size of used area. Predicted values are from log_10_ transformed data.

**Figure 2:**
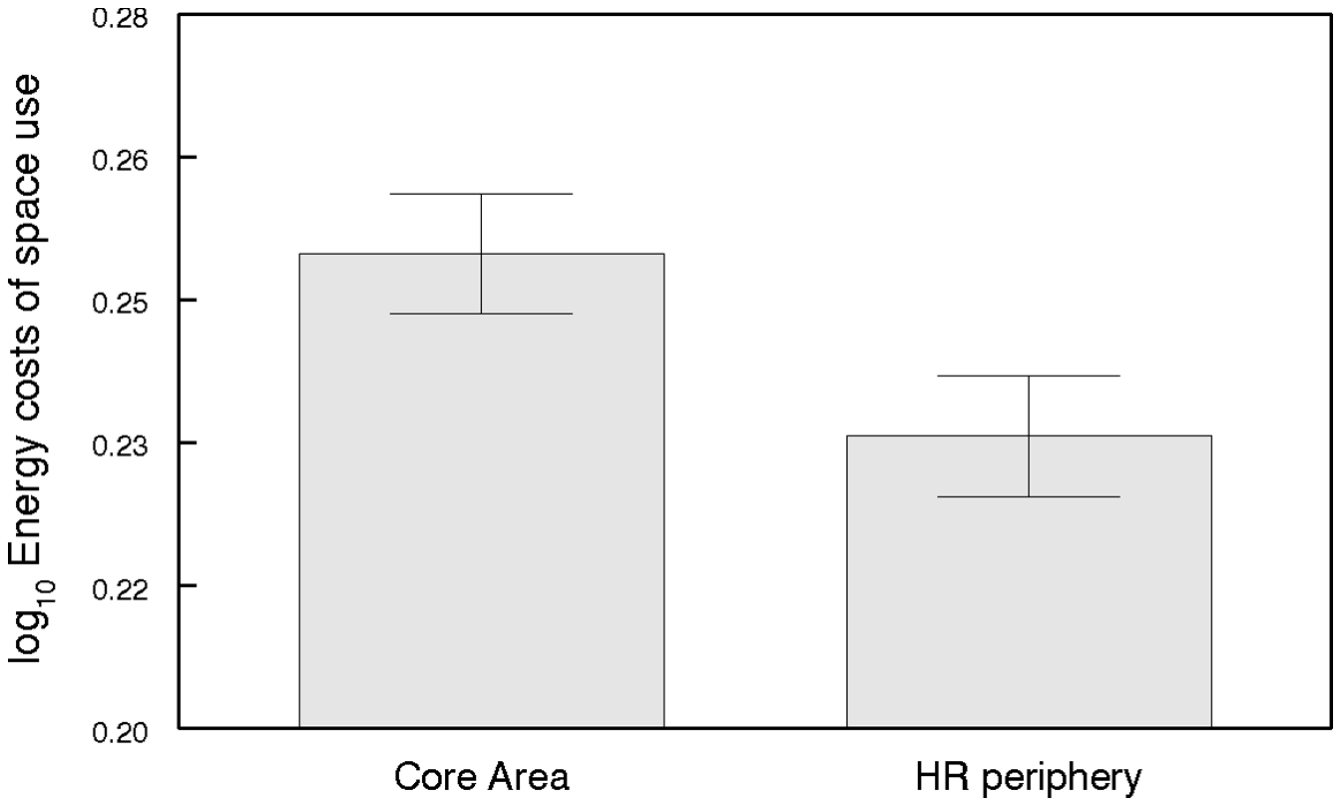
Energy costs of space use within the CA and the HR periphery. Adjusted means are from log_10_ transformed data.

Final LMM model contained the fixed factors used area and fish position for energy costs of space use analyses. Details of the model are shown in Tables 2 and 3. Differences in energy costs of space use with regard to the size of the used area were observed. The energy spent by catfish decreased with increasing size of the used area (Fig. 1). In other words, energy costs of space use within a small area were higher. As further documented, catfish spent significantly more energy within the CA compared to the HR periphery (Fig. 2) and were present in the CA, delineated by 50% contour of the HR, for 77% of their locations. Thus, the CA was suggested as an important area where catfish spent most of their temporal and energy budget.

**Table 2:** Solution for fixed effects (parameter estimates with corresponding standard errors and p-values for the final LMM model).

Parameter	Factor level	Estimate	Sd. Error	P<
Intercept	-	0.2853	0.02176	0.0001
Used area	-	−0.01582	0.00579	0.0069
Fish position	CA	0.02038	0.00805	0.0121
	HR periphery	0	0	-

**Table 3:** Type 3 tests of fixed effects for final LMM model.

Effect	Num DF	Den DF	F	P<
Used area	1	201	7.45	0.0069
Fish position	1	214	6.41	0.0121

## Discussion

The results of our study revealed that the catfish that occurred within a large area consumed less energy than those that used a small area. It is generally known that animals can satisfy their energy needs within small areas, provided that sufficient resources are available there [41]. For example, a predator occupying a large HR, such as the lynx *Lynx rufus*, reduces size of the area used as food density increases [19]. Similarly, a CA containing a high density and diversity of feeding trees is preferred over non-core areas by the highly mobile spider monkey *Ateles geoffroyi* [17]. Therefore, we can assume that high degree of catfish energy costs in small areas indicates the use of limited resources allocated within that small region. Accordingly, freshwater herbivorous grass carp, *Ctenopharyngodon idella*, were associated with submerged vegetation 50% of time in 16% of the HR area [42]. In addition, collared lizards, *Crotaphytus collaris*, were present in approximately 25% of the HR 66% of the time they were observed [43].

The use of an area and energy costs of an individual are also considered in relation to their defense of a preferred area when the residents exclusively occupy the CA [11]. It is generally known that defended areas where residents pay higher energy costs to maintain exclusivity appeared to be smaller than non-defended areas [44]. The results of our study generally indicate that catfish defended energetically optimal areas rather than a large HR area, as indicated by the inverse relationship between the size of the used area and the associated energy costs. Our findings are in agreement with the previous studies [26], where the catfish energy costs increased when the CAs of conspecifics overlapped and both of them were inside CAs simultaneously. Large catfish aggregations were visually observed in the wild within small areas [45]. Aggregating can provide various benefits as well as costs resulting from sharing resources [46]. One of the costs is interference [47], which does not necessarily mean an overt aggression. In aggregations of another large fish predator, pike *Esox lucius* (L.) was suggested interference operated through intimidation [48]. Stress related to intimidations could also be the reason of increased catfish energy costs within the CA. In addition to feeding and defense, other activities associated with the movement of species within the HR can be considered. For example, low energy costs of movement were reported for fish from the Labridae family moving within a large area, and the activity was expected to represent the testing of a social situation as opposed to active food intake [49]. The movement of animals within the HR appeared to be related to low-energy activities, such as patrolling and searching for new resources and/or partners, whereas energetically demanding movement within small areas appeared to be related to the use of limited resources.

Low amounts and wide dispersal of available resources induce an increase in the HR [2], [19]. Apparently, an increase in movement activity can be expected in the search for these resources [41]; however, these HR relocations can be energetically less demanding than the resources use itself [49]. Similarly, intensive movement does not always imply a large HR for an individual [50]. The aquatic environment allows fish to move with minimal energy expenditures; however, energy expenditures rapidly increases during active movement [33]. Furthermore, our results revealed differences in degree of energy costs with regard to the size of the area used. Hence, we can infer that CA is of the special importance as a source of critical resources. We can conclude that energy costs and the size of the area used are not necessarily positively correlated; sufficient resource availability allows animals to use small areas where the majority of energy is located and consumed.
